# Peripheral Opioid Antagonist Enhances the Effect of Anti-Tumor Drug by Blocking a Cell Growth-Suppressive Pathway *In Vivo*

**DOI:** 10.1371/journal.pone.0123407

**Published:** 2015-04-08

**Authors:** Masami Suzuki, Fumiko Chiwaki, Yumi Sawada, Maho Ashikawa, Kazuhiko Aoyagi, Takeshi Fujita, Kazuyoshi Yanagihara, Masayuki Komatsu, Minoru Narita, Tsutomu Suzuki, Hiroshi Nagase, Ryoji Kushima, Hiromi Sakamoto, Takeo Fukagawa, Hitoshi Katai, Hitoshi Nakagama, Teruhiko Yoshida, Yasuhito Uezono, Hiroki Sasaki

**Affiliations:** 1 Division of Cancer Pathophysiology, National Cancer Center Research Institute, Tokyo, Japan; 2 Division of Genetics, National Cancer Center Research Institute, Tokyo, Japan; 3 Division of Cancer Development System, National Cancer Center Research Institute, Tokyo, Japan; 4 Department of Translational Oncology, National Cancer Center Research Institute, Tokyo, Japan; 5 Division of Translational Research, Exploratory Oncology Research & Clinical Trial Center, National Cancer Center Hospital East, Chiba, Japan; 6 Department of Pharmacology, Hoshi University School of Pharmacy and Pharmaceutical Sciences, Tokyo, Japan; 7 Department of Toxicology, Hoshi University School of Pharmacy and Pharmaceutical Sciences, Tokyo, Japan; 8 International Institute for Integrative Sleep Medicine, University of Tsukuba, Tsukuba, Japan; 9 Department of Pathology, National Cancer Center Hospital, Tokyo, Japan; 10 Gastric Surgery Division, National Cancer Center Hospital, Tokyo, Japan; University of Navarra, SPAIN

## Abstract

The dormancy of tumor cells is a major problem in chemotherapy, since it limits the therapeutic efficacy of anti-tumor drugs that only target dividing cells. One potential way to overcome chemo-resistance is to “wake up” these dormant cells. Here we show that the opioid antagonist methylnaltrexone (MNTX) enhances the effect of docetaxel (Doc) by blocking a cell growth-suppressive pathway. We found that PENK, which encodes opioid growth factor (OGF) and suppresses cell growth, is predominantly expressed in diffuse-type gastric cancers (GCs). The blockade of OGF signaling by MNTX releases cells from their arrest and boosts the effect of Doc. In comparison with the use of Doc alone, the combined use of Doc and MNTX significantly prolongs survival, alleviates abdominal pain, and diminishes Doc-resistant spheroids on the peritoneal membrane in model mice. These results suggest that blockade of the pathways that suppress cell growth may enhance the effects of anti-tumor drugs.

## Introduction

Chemoresistance is often observed in tumor therapy, and can lead to a poor prognosis. One potential mechanism of such resistance is the arrest of tumor cell division (i.e., a dormant state), which would enable cells to escape attack by chemotherapeutic reagents that only affect dividing cells [1–3]. Thus, the usual chemotherapy for these tumors may lead to regression, but seldom to a cure. By targeting these dormant residual tumor cells, we may be able to overcome chemoresistance through the development of reagents that can enhance the effectiveness of currently-available anti-tumor drugs.

Gastric cancer (GC) is the second-leading cause of cancer-related mortality [4]. There are two histopathological categories, intestinal-type (well- or moderate-differentiated) and diffuse-type (poorly-differentiated and signet ring cell), which have distinct pathogenesis and genetic profiles [5, 6]. In intestinal-type GCs, the tumor cells adhere to each other, and tend to arrange themselves in tubular or glandular formations. In contrast, a lack of adhesion molecules in diffuse-type GCs allows the individual tumor cells to grow and invade neighboring structures [5, 6]. Diffuse-type GC has the potential to disseminate and grow in the peritoneal cavity. This condition is often associated with disease progression and a poor prognosis [7]. Overall survival in patients with peritoneal dissemination is only slightly influenced by systemic chemotherapy, so that the occurrence of peritoneal dissemination is regarded as a terminal condition in GC patients. Accordingly, more effective treatments are needed.

Opioid growth factor (OGF, also known as Met-enkephalin) is an endogenous opioid that has been reported to suppress cell growth by binding to OGF receptor (OGFR) in some cancers [8, 9]. The mechanism of OGF-induced cell growth suppression is related to the cyclin-dependent kinase inhibitory pathway [9]. This biological effect of OGF is reversible, non-cytotoxic and non-apoptotic to tumor cells [9]. Although the molecular structure of OGFR has no homology to that of classical opioid receptors, the biological effects of OGF can be blocked by opioid antagonist [9].

In the present study, we found that OGF is over-expressed in diffuse-type GCs. Moreover, the combined use of the peripheral opioid antagonist methylnaltrexone (MNTX), which is used to manage opioid-induced constipation, and the chemotherapeutic agent docetaxel (Doc) diminishes Doc-resistant spheroids on the peritoneal membrane followed by the inhibition of micrometastasis and an increase in survival time in peritoneal-dissemination model mice. Our findings suggest that the strategy of awakening and killing tumor cells has potential for resolving the major problem of dormancy of tumor cells and overcoming the development of peritoneal dissemination.

## Materials and Methods

### Human tissues and patient’s ascites

All of gastric cancer (GC) tissues and patient’s ascites was provided by the National Cancer Center Hospital after obtaining written informed consent from each patient and approval by National Cancer Center Institutional Review Board (ID: No.17-030). All cancer specimens were reviewed and classified histopathologically according to the Japanese Classification of Gastric Cancer. Tissue specimens were immediately frozen with liquid nitrogen after surgical extraction, and stored at -80°C until use.

### Animals

Six-week-old female C.B17/Icr-scid mice were used. Mice were purchased from CLEA Japan (Tokyo, Japan) and housed at a room temperature of 23 ± 1°C with a 12 h light/dark cycle. The mice were maintained under specific pathogen-free conditions and provided sterile food, water, and cages. All experiments were conducted in accordance with the ethical guidelines of the International Association for the Study of Pain and were approved by the Committee for Ethics in Animal Experimentation of the National Cancer Center. Efforts were made to minimize the numbers and any suffering of animals used in the subsequent experiments.

### Immunochemistry

Specimens fixed in formalin and embedded in paraffin were cut into 5 μm sections, subsequently dewaxed, and dehydrated. Sections were blocked for DAKO protein block (DAKO, Carpinteria, CA), and stained with a primary antibody against Ki-67 antigen (1:75; DAKO) at room temperature for 1 h. Subsequently, the sections were subjected to DAB (substrate buffer + DAB chromogen [× 50]) for 5 min. The slides were counterstained with hematoxylin and then mounted.

### Microarray analysis

Total RNA was isolated by suspending the cells in an ISOGEN lysis buffer (Nippon Gene, Toyama, Japan) followed by precipitation with isopropanol. We used Human Expression Array U95A version 2 (Affymetrix, Santa Clara, CA) for analysis of mRNA expression levels corresponding to 12,600 transcripts. The procedures were conducted according to the suppliers’ protocols. The expression value (average difference: AD) of each gene was calculated using GeneChip Analysis Suite version 4.0 software (Affymetrix). The mean of AD values in each experiment was 1000 to reliably compare variable multiple arrays. Hierarchical clustering is widely used as one of the unsupervised learning methods. Hierarchical clustering of microarray data was performed by the use of GeneSpring (Agilent Technologies Ltd., Palo Alto, CA), Microsoft EXCEL, and Cluster & TreeView software [29]. All the microarray data have been deposited in a MIAME compliant database, GEO; the accession number GSE47007. By Wilcoxon u-test (*p*<0.05) and a 2-fold change, 188 genes were selected as specific genes for 18 intestinal-type GCs, and 704 genes were selected as specific genes for 12 diffuse-type GCs. The results of a two-dimensional hierarchical clustering analysis of the 892 selected genes are shown.

### Reverse transcription polymerase chain reaction (RT-PCR)

Total RNA was isolated from tissues or suspending cells using ISOGEN lysis buffer (Nippon Gene), followed by precipitation with isopropanol. As described in our previous report [29], semi-quantitative RT-PCR was carried out using primer sets (Table S1). For semi-quantitative RT-PCR, we showed data within linear range by performing 25–30 cycles of PCR.

### Cell lines and primary culture

A human diffuse-type GC derived-cell line, HSC-60 was established [10]. A highly peritoneal-seeding cell line, 60As6 was established from HSC-60. Other human GC cell lines (HSC-39, HSC-42, HSC-43, HSC-44, 44As3, HSC-58, 58As1, 58As9, HSC-59, SNU-16, KATOIII, MKN45, TMK-1, OKAJIMA and PANC-1) and mouse fibroblast cell line (NIH3T3) were also used. Of them, nine cell lines (HSC-39, HSC-42, HSC-43, HSC-44, 44As3, HSC-58, 58As1, 58As9, HSC-59) were established by a collaborator, Dr. Yanagihara; three cell lines (SNU-16, PANK-1, NIH3T3) and two cell lines (KATOIII, MKN45) were purchased from American Type Culture Collection and Japanese Collection of Research Bioresources Cell Bank, respectively. The other cell lines (TMK-1 and OKAJIMA) were obtained from our collaborators in Japan. A mouse peritoneum-derived cell line, 1Cs-mM was originally established. Primary cultured GC cells (NSC-16C) were obtained from diffuse-type GC patient’s ascites. The ascites was centrifuged and the pellet was suspended. All those cell lines and a primary culture were maintained in an RPMI1640 medium supplemented with 10% fetal bovine serum, 100 IU/mL penicillin G sodium, and 100 μg/ml streptomycin sulfate under an atmosphere of 5% CO_2_ and 95% air at 37°C. To establish transfectants that expressed the luciferase or GFP gene, a transfection reagent, LipofectAMINE 2000 (Invitrogen, Carlsbad, CA) was used. Geneticin (500 μg/ml; Invitrogen) was used to select stable transfectants, and transfected clones were screened for luciferase gene expression by detecting bioluminescence using an IVIS system (Xenogen, Alameda, CA). Clones that expressed the luciferase or GFP gene were referred to as 60As6-Luc cells or 60As6-GFP cells.

### Drug preparation

[Met^5^]-enkephalin (OGF) was purchased from Wako (Tokyo, Japan). Methylnaltrexone (MNTX) was provided from Drs. H. Nagase & T. Suzuki. OGF and MNTX were dissolved in sterile water or saline. Docetaxel (Doc) was purchased from Aventis Pharma Co., Ltd. (Tokyo, Japan). Doc was prepared according to the manufacturer’s instructions and diluted with sterile water or saline.

### OGFR shRNA-bearing lentiviral particles transduction

Cells were cultured at a density of 1 x 10^2^ cell per well in 24-well plates. After 24 h, cells were transferred to a medium containing Polybrene (Santa Cruz Biochemistry, Santa Cruz, CA) at a final concentration of 5 μg/ml, and we then added the *OGFR* shRNA lentivial particles (Santa Cruz Biochemistry) at MOI: 8 and incubated overnight. The medium with lentivial particles was removed, and the cells were cultured in a normal medium without polybrene. After 48 h, the cells were replaced with selection medium containing 2 μg/ml Puromycin, and then several colonies were picked up.

### Cell growth assays

OGF-induced cell growth inhibition was determined by a cell proliferation assay using MTT assay (1 x 10^3^ cells/well, 96-well plates). OGF (10^-4^ M), combinations of OGF and MNTX (10^-6^ M) or sterile water were added beginning 24 h after seeding. Both media and compounds were replaced daily. Seventy-two hours after treatment, 20 μl of 5 mg/ml MTT solution was added to each well of the culture medium. After incubation for an additional 4 h, the medium was removed and 100 μl of DMSO was added to resolve the formazan crystals. Optical density was measured using a microplate reader with an absorption wavelength of 563 nm. In each experiment, three replicates were prepared for each sample. The proportion of cell growth was determined based on the difference in absorbance between the samples and controls. MNTX-induced cell growth was conducted in 24-well plates (60As6: 2 x 10^4^ cells/well and HSC-42: 1 x 10^4^ cells/well) under normal nutrient (10% FBS) and low nutrient (2% FBS) conditions. Twenty-four hours after seeding, cells were treated with MNTX (10^-6^ and 10^-5^ M) or sterile vehicle for 72 h. Cells were harvested with a solution of 0.05% trypsin/0.53 mM EDTA, centrifuged, and counted with TC20 Automated Cell Counter (Bio-Rad, Hercules, CA). Doc-induced cell growth inhibition was conducted in 6-well plates (60As6: 2 x 10^5^ cells/well and HSC-42: 5 x 10^4^ cells/well). Twenty-four hours after seeding, cells were treated with Doc (10^-9^ M) or sterile vehicle for 48 h, and subsequently treated with Doc, combinations of Doc and MNTX (10^-6^ M), or a sterile vehicle for 48 h. Cells were harvested with a solution of 0.25% trypsin/0.53 mM EDTA, centrifuged, and counted with a hematcytometer. Cell viability was determined by trypan blue staining.

### Co-culture and cell growth assays

1Cs-mM and NIH3T3 cells were plated in 24-well plates (2 x 10^4^ cells/well). Twenty-four hours after plating, 60As6-GFP cells (1 x 10^4^ cells/well) were seeded on 1Cs-mM and NIH3T3 cells respectively, and treated with MNTX (10^-5^ M) or sterile water for 72 h. Cells were harvested with a solution of 0.25% trypsin/0.53 mM EDTA, centrifuged, and counted with a hematcytometer under a fluorescence microscope TS100 (Nikon, Tokyo, Japan).

### Tumor cell inoculation

The density of 60As6-Luc or 60As6-GFP cells was adjusted to 1 × 10^6^ cells per 1 ml phosphate-buffered saline (PBS). In the experimental group, the cell suspension was injected into the abdominal cavity *via* a 26 1/2-gauge needle inserted into the central abdomen. In the control group, PBS was injected into the abdominal cavity instead of tumor cells.

### Tumor growth and survival studies

The day of tumor cell inoculation was considered day 0. Mice were weighed and measured weekly for tumor growth. For that measurement, whole-body luciferase imaging with an IVIS imaging system was used to visualize the 60As6-Luc cells, as described previously [22]. *In vivo* photon counting analysis was determined using Living Image software (version 2.50) (Xenogen). On day 7 after tumor inoculation, mice were divided evenly into 4 groups based on the number of photon counts. Mice were then treated intraperitoneally with saline, MNTX (0.3 mg/kg), Doc (0.5 mg/kg) and Doc/MNTX twice weekly until endpoint criteria were met. Endpoint criteria included severe cachexia, significant weight loss exceeding 20% of initial weight, or extreme weakness or inactivity.

### Assessment of metastatic tumor cells

An *in vivo* imaging system OV110 (Olympus, Tokyo, Japan) was used to observe 60As6-GFP cells attached to the mesentery. Forty-nine days after the inoculation, mice were deeply anesthetized with isoflurane and the intestine and mesentery were quickly removed after decapitation. To analyze the number of single cells or spheroids on the mesentery and peritoneal surface, we captured 10 images per each intestine and mesentery.

### Behavioral test

Hunching behavior was examined as described previously with some modifications [30]. Briefly, mice were placed individually in the center of an open field arena and observed for 180 sec. The hunching score was the total time (sec) that the mouse exhibited hunching behavior multiplied by the scoring factor, which was defined according to our recent paper [31]: 0 = normal coat luster, displays exploratory behavior. 1 = mild rounded-back posture, displays slightly reduced exploratory behavior, normal coat luster. 2 = severe rounded-back posture, displays considerably reduced exploratory behavior, piloerection, intermittent abdominal contractions. Behavioral testing was performed on day 35 after tumor inoculation.

### Western blotting

Cells were lysed in RIPA buffer containing 1% protease inhibitor cocktail. The protein concentration of each sample was measured using a BCA protein assay kit (Thermo Fisher Scientific, Rockford, IL). Whole cell lysates were subjected to 15% SDS-PAGE using standard protocols. The following antibodies were used: p21 (BD Pharmingen, San Diego, CA), OGFR (Proteintech, Chicago, IL), β-actin (Cell Signaling Technology, Danvers, MA), and glyceraldehyde 3-phosphate dehydrogenase (GAPDH) (Santa Cruz Biochemistry). Membranes were probed with secondary anti-rabbit or anti-mouse horseradish peroxidase-conjugated antibodies, and developed using a chemiluminescence Western blotting detection system SuperSignal (Thermo Fisher Scientific).

### ELISA

OGF level of ascites and peritoneal washings obtained from patients or mice with peritoneal dissemination were assessed by an EIA kit (Peninsula Labs, San Carlos, CA) according to the manufacturer’s instructions. All samples were studied in duplicate.

### Flow cytometry

Cells were transferred to serum-free medium for 96 h, and subsequently harvested with 0.05% trypsin-EDTA, and then suspended in cold 80% ethanol. After fixation at -20°C overnight, the samples were washed in PBS (-) and incubated in 500 μl of PI/RNase Staining Buffer (BD Pharmingen) per 1 x 10^6^ cells for 30 minutes at room temperature before analysis. Flow cyctometry was performed using FACSCalibur (BD, Franklin Lakes, NJ), and analyzed by a software, FlowJo (Tree Star, Inc., Ashland, OR).

### siRNA transfection

*OGFR* siRNA was introduced to 60As6 cells using Dharma FECT1 (Dharmacon, La Fayette, CO), following the procedure recommended by the manufacturer. RT-PCR and Western blot analyses were carried out after the experiment.

### Automated image capture and cell cycle analysis

60As6 cells were seeded at 1 x 10^3^ cells per well on 96-well plates the day before treating them with OGF (10^-6^–10^-4^ M). Forty-eight hours after treatment, 5-ethynyl uridine (EdU) was added for 6 h and stained with EdU antibody. Cell nuclei were labeled with Hoechst 33342. The DNA content and EdU intensity were quantitatively analyzed by the Cellomics ArrayScan high content microscope with the Cell Cycle BioApplication software (Thermo Fisher Scientific).

### Statistics

Statistical analyses were carried out using GraphPad Prism version 5.0a (GraphPad Software, La Jolla, CA). The statistical significance of cell growth data was assessed with one-way ANOVA followed by the Bonferroni multiple comparisons test (cell growth) or two-tailed unpaired Student *t* test (for co-culture, micrometastasis and behavioral test). Mortality data was compared using Kaplan-Meier plots and Log-rank test. A *p* value of<0.05 was considered statistically significant.

## Results

### Expression of *PENK* encoding OGF in diffuse-type GCs

**Fig 1:**
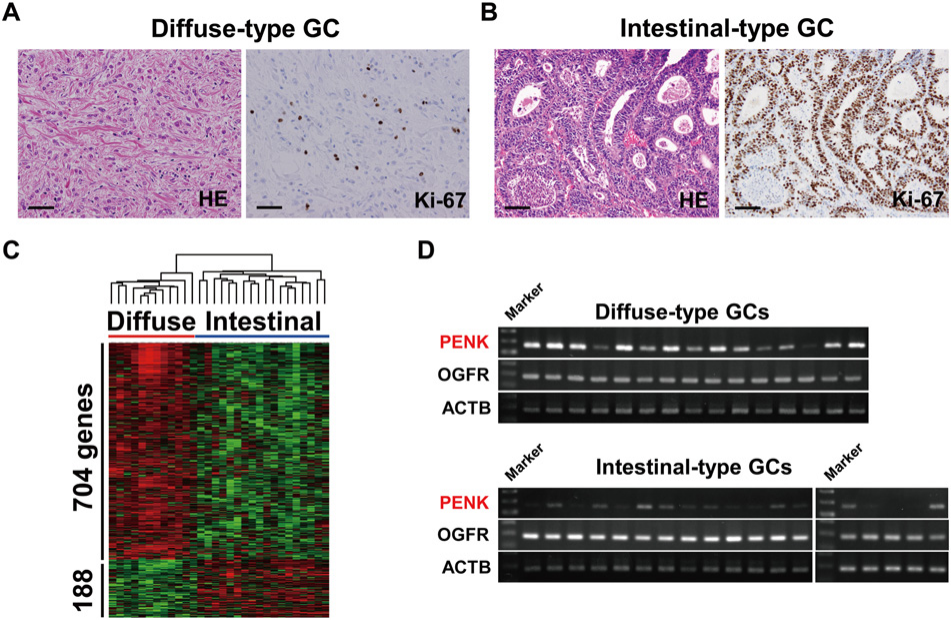
*PENK* encoding opioid growth factor (OGF) is preferentially expressed in diffuse-type gastric cancers (GCs). Representative histological image (hematoxylin-eosin, HE) and Ki-67 immunostaining of diffuse-type GC (A) and intestinal-type GC (B). Scale bar, 50 μm. C, supervised clustering analysis of 892 specifically expressed genes in 12 diffuse-type and 18 intestinal-type GCs. By the Wilcoxon u-test (*p*<0.05) and a 2-fold change, 188 genes were selected as specific genes for 18 intestinal-type GCs, and 704 genes were selected as specific genes for 12 diffuse-type GCs. The results of a two-dimensional hierarchical clustering analysis of the 892 selected genes are shown. D, RT-PCR analyses of OGF signaling molecules, *PENK* and its receptor *OGFR*, in diffuse-type and intestinal-type GCs.

**Fig 2:**
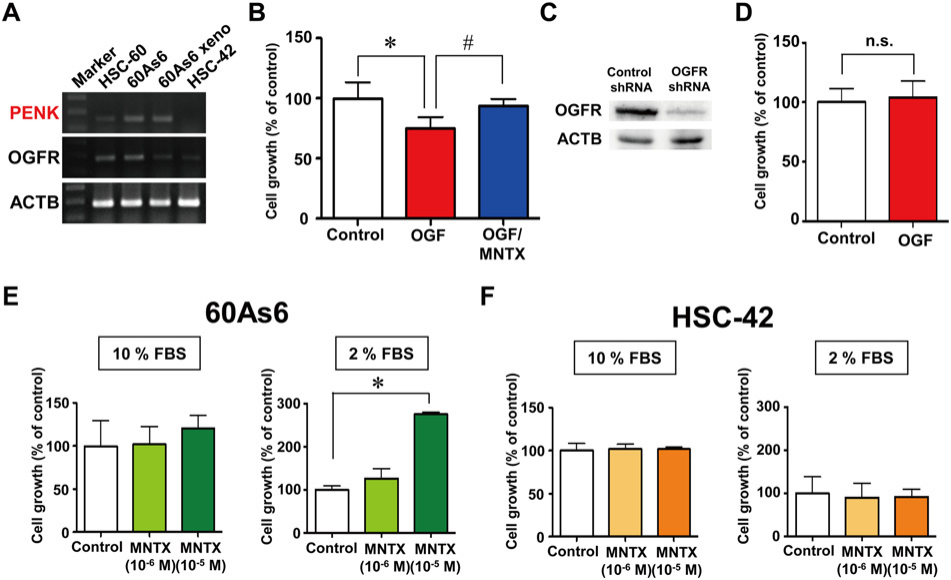
Blockade of OGF signaling by methylnaltrexone (MNTX) increased the growth of a diffuse-type GC cell line, but not an intestinal-type GC cell line, under low nutrient conditions. A, RT-PCR analyses of *PENK* and *OGFR* in diffuse-type GC cell lines, HSC-60 cells and highly metastatic 60As6 cells, 60As6 xenograft (60As6 xeno) and the intestinal-type GC cell line HSC-42. B, growth of 60As6 cells treated with OGF (10^-4^ M), methylnaltrexone (MNTX, 10^-6^ M), or a combination of these compounds for 72 h (mean ± SD, n = 3–6 per group, *p<0.05, control vs. OGF, #*p* <0.05, OGF vs. OGF/MNTX). C, western blot analysis of OGFR protein in 60As6 cells with a stable transfectant of *OGFR* shRNA or control shRNA. D, growth of the stable transfectant of *OGFR* shRNA of 60As6 cells in the presence or absence of OGF (10^-4^ M) for 72 h. Non-targeting control shRNA was used as a control (mean ± SD, n = 3 each). Growth of 60As6 cells (E) and HSC-42 cells (F) treated with MNTX (10^-6^ and 10^-5^ M) or a vehicle for 72 h under normal nutrient (10% FBS) and low nutrient (2% FBS) conditions. (mean ± SD, n = 4 each, *p<0.05, vs. control).

Solid tumors with diffuse growth are composed of many myofibroblasts and few vessels (e.g., pancreatic cancers and scirrhous type of breast cancer) (S1 Fig). Depending on the conditions related to the microenvironment, such as nutrient deficiency, these tumors show a high prevalence of rarely-proliferative tumor cells. In support of these reports, diffuse-type GC shows a high proportion of Ki-67-negative non-proliferating tumor cells compared to intestinal-type GC (Fig 1A and 1B). We previously established a highly peritoneal metastatic cell line 60As6 by the 6-times transplantation of a diffuse-type GC-derived parental cell line (HSC-60) into a mouse peritoneal cavity [10]. We found that 60As6 cells exhibit apparent resistance to Doc by inducing growth arrest in G1 phase under serum starvation (S2A–S2C Fig), suggesting that the acquisition of this characteristic might be attributed to a particular tumor microenvironment, such as hyponutrition. To address the molecular mechanisms of dormancy related to chemoresistance of diffuse-type GC, we first searched for cell growth-suppressive signal pathways by a comparative gene expression analysis between 12 primary diffuse-type and 18 intestinal-type GCs (Fig 1C). In most of the intestinal-type GCs, *CDC6*, which is a typical marker of S-phase progression in the cell cycle, was highly expressed (S3A Fig). On the other hand, *PENK* encoding OGF was identified to be over-expressed in diffuse-type GCs (Fig 1C). *PENK* mRNA was also confirmed to be preferentially expressed in diffuse-type GCs compared with intestinal-type GCs by RT-PCR (Fig 1D). An increase in OGF secretion in cancer-associated ascites, but not peritoneal washings, was confirmed by ELISA (S3B Fig). Consistent with the expression profile of primary GCs, *PENK* and *OGFR* were expressed in the diffuse-type GC cell line HSC-60, 60As6 cells, and their mouse xenografts (60As6 xeno), whereas the intestinal-type GC cell line HSC-42 expressed only *OGFR* mRNA (Fig 2A). OGF has been reported to bind to two membrane receptors, μ- and δ-opioid receptors (*OPRM1* and *OPRD1*, respectively), as well as to the nuclear receptor OGFR. Quite low expression of *OPRM1* and *OPRD1* was detected in both HSC-60 and 60As6 cells (S4A Fig). In various diffuse-type GC cell lines, little or no expression of these two membrane receptors was detected by RT-PCR (data not shown), whereas high expression levels of both *PENK* and *OGFR* were found in most of these cell lines (Fig S4B).

### Blockade of OGF signaling by MNTX increased the cell growth of a diffuse-type GC cell line under low nutrient conditions

The growth of 60As6 cells treated with OGF, the opioid antagonist MNTX or both was measured by the MTT assay. Treatment with OGF for 72 h significantly inhibited cell growth (69.4 ± 9.8%), which was clearly restored by MNTX (93.4 ± 6.0%, Fig 2B). To investigate the mechanism of OGF-induced cell growth inhibition, a cell population of 60As6 cells after treatment with OGF was acquired from the ArrayScan HCS reader. Cells in different cell-cycle phases G1, S and G2/M were separated by a cell population analysis according to ErdU incorporation and DNA content. Treatment with OGF for 48 h resulted in a dose-dependent increase in the G1 population of 60As6 cells (S5A Fig). Accordingly, the number of cells at the G2/M phase was decreased. Furthermore, this effect was significantly diminished by MNTX (S5B Fig). It has been reported that the expression of p16 and/or p21 is related to OGF-induced G1-arrest in cancers of the head and neck, ovaries, and pancreas [11–13]. In 60As6 cells, p21 protein was evidently increased at 3 h after treatment with OGF (S5C Fig), whereas p16 protein was not detected due to homozygous deletion of the gene (data not shown). With the use of two different RNA-interference experiments, we confirmed that OGFR is required for the suppression of cell growth by OGF. The growth of *OGFR* siRNA-transfected cells was not suppressed by OGF (S5D and S5E Fig), and a stable transfectant of *OGFR* shRNA, in which OGFR protein was reduced (Fig 2C), was also not affected by OGF (Fig 2D). We then examined the effect of the inhibition of OGF signaling by MNTX on cell growth under both normal and low nutrient conditions (10% or 2% FBS, respectively). In the low nutrient condition, treatment with MNTX for 72 h clearly increased the growth of 60As6 cells (10^-6^ M: 126.4 ± 46.7%, 10^-5^ M: 275.6 ± 10.1%, Fig 2E), but not HSC-42 cells (10^-6^ M: 90.0 ± 33.6%, 10^-5^ M: 92.2 ± 16.5%, Fig 2F). Cultures that were treated with MNTX under the normal nutrient condition showed no differences in the numbers of both cell lines compared to the control (Fig 2E and 2F).

### Blockade of OGF released from mesothelial cells by MNTX increased the growth of diffuse-type GC cells in a co-culture system with mesothelial cells

**Fig 3:**
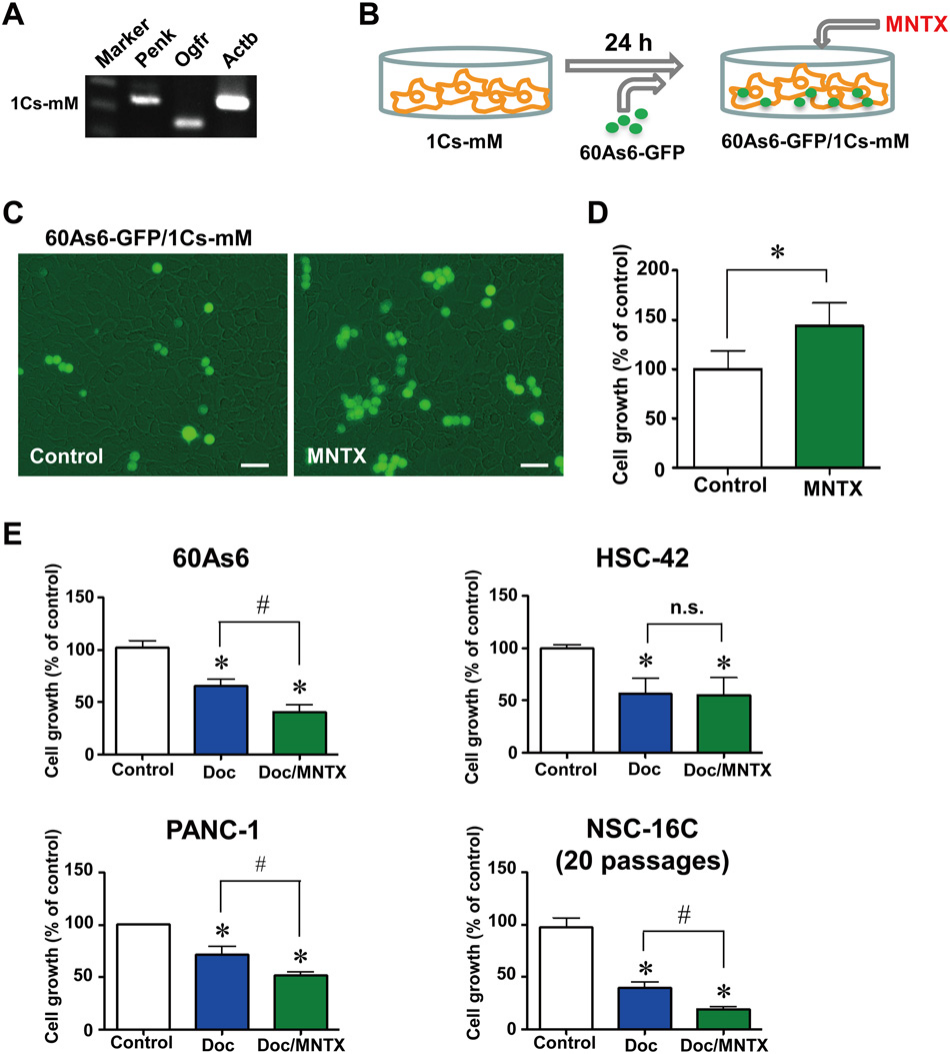
Blockade of OGF signaling by MNTX increased the growth of diffuse-type GC cells co-cultured with mesothelial cells. A, RT-PCR analyses of *Penk* and *Ogfr*, in mouse mesothelial cells (1Cs-mM). B, schematic illustration of the system for co-culture of 60As6-GFP and 1Cs-mM. C, Growth of 60As6-GFP cells co-cultured with 1Cs-mM cells in the presence or absence of MNTX (10^-5^ M) for 72 h. Scale bar, 20 μm. D, the growth of 60As6-GFP cells was calculated (mean ± SD, n = 3 each, *p<0.05). E, growth of the diffuse-type GC cell line 60As6 cells, the intestinal-type GC cell line HSC-42 cells, the pancreatic cancer cell line PANC-1 cells, and primary cultured GC cells derived from the ascites of a patient NSC-16C cells treated with Doc (10^-9^ M) or a vehicle for 48 h, and subsequently treated with Doc, Doc/MNTX (10^-6^ M) or a vehicle for 48 h. Cells were counted with a hemacytometer (mean ± SD, n = 4 each, *p<0.05, vs. control, #*p*<0.05, Doc vs. Doc/MNTX).

The microenvironment for the peritoneal metastasis of diffuse-type GC is made up of mesothelial cells and myofibroblasts [14–16]. The first surface that free tumor cells encounter is the innermost layer of the peritoneum, the mesothelium. The mesothelium forms a cellular monolayer supported by a basement membrane. The adherence of tumor cells to the mesothelium is the second step in the metastatic cascade, which temporarily arrests the tumor cells to their eventual site of metastasis [17]. A recent study suggested that these cells are functionally organized to promote the survival of tumor cells in the host [18]. Interestingly, our originally established mouse mesothelial cell line 1Cs-mM (Fig 3A) also expresses *Penk* mRNA. Thus, we next investigated whether OGF released by mesothelial cells suppressed tumor cell growth using a co-culture cell system of GFP-expressing 60As6 (60As6-GFP) cells and a mouse mesothelial cell line, 1Cs-mM cells (Fig 3B). Twenty-four hours after 1Cs-mM cells were plated, 60As6-GFP cells were seeded at a 1: 4 (60As6-GFP: 1Cs-mM) ratio and treated with MNTX or vehicle for 72 h. As expected, treatment with MNTX significantly increased 60As6-GFP cell growth in this system (144.0 ± 23.3%, Fig 3C and 3D), indicating that peritoneal mesothelium-derived OGF can also arrest tumor cell growth. Myofibroblasts or carcinoma-associated fibroblasts are the most abundant cell type in the primary tumor and have been shown to promote resistance to anti-tumor drugs [19]. The mouse fibroblast cell line NIH3T3 also expresses *Penk* mRNA (S6A Fig). As with mesothelial cells, treatment with MNTX was associated with a significant increase in the growth of 60As6-GFP cells in the co-culture system with NIH3T3 cells (122.5 ± 12.6%, S6B and S6C Fig).

### Blockade of OGF signaling by MNTX boosts the anti-tumor effect of Doc *in vitro*

We next evaluated the effect of MNTX on the Doc-induced inhibition of cell growth. In both 60As6 and HSC-42 cells, treatment with Doc alone and Doc/MNTX obviously suppressed cell growth relative to that in vehicle-treated cells (Fig 3E). Notably, the combination of MNTX with Doc significantly decreased cell growth compared with Doc alone in 60As6 cells (Doc: 65.3 ± 6.6%, Doc/MNTX: 40.5 ± 7.1%, Fig 3E). In contrast, this booster effect of MNTX was not observed in HSC-42 cells (Doc: 56.0 ± 15.0%, Doc/MNTX: 55.0 ± 16.7%), in which *PENK* expression was not detected (Fig 3E). MNTX had the same effect on the pancreatic cancer cell line PANC-1 (Doc: 71.3 ± 8.1%, Doc/MNTX: 51.3 ± 4.2%, Fig 3E), and pancreatic cancers often express OGF and are well known to show diffuse growth (S1 Fig). We further investigated the effect of the combination of MNTX and Doc on primary cultured GC cells (NSC-16C) derived from the ascites of a patient with diffuse-type GC. The results showed that MNTX clearly boosted the anti-tumor effect of Doc in this clinical subject as well as in a GC cell line with a high level of *PENK* expression (Doc: 39.2 ± 5.8%, Doc/MNTX: 19.2 ± 2.1%, Fig 3E).

### Combined use of Doc and MNTX significantly prolongs survival in peritoneal dissemination model mice

**Fig 4:**
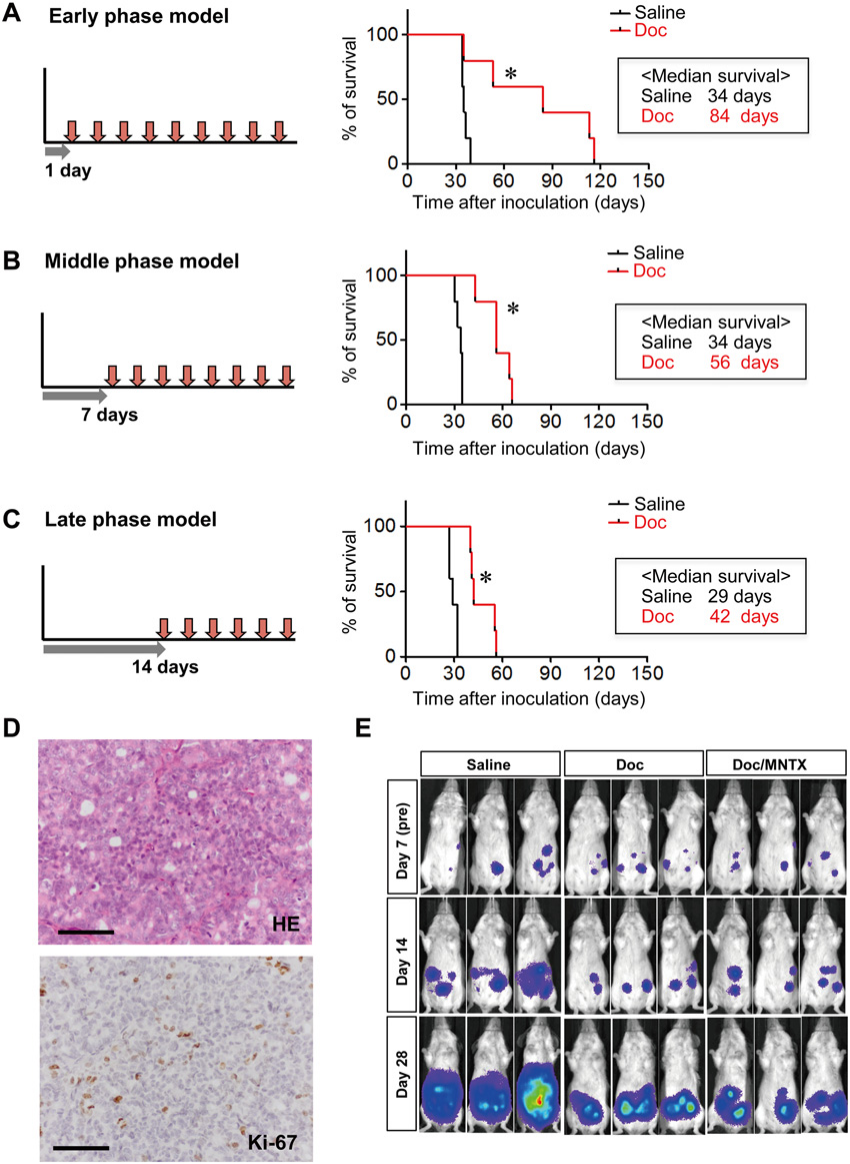
Mouse models of intraperitoneal low-dose Doc therapy corresponding to 3 different phases (early, middle, and late) in the progression of peritoneal dissemination. Survival curves for the early phase (A), middle phase (B), and late phase (C) of a peritoneal metastasis model. Administration of Doc (0.5 mg/kg) was started 1 day (A), 7 days (B) or 14 days (C) after the inoculation of 60As6-Luc cells, and was continued until the endpoint criteria were reached (n = 5, *p<0.05, vs. saline). D, representative histological image (hematoxylin-eosin, HE) and Ki-67 immunostaining of 60As6 xenograft. Scale bar, 50 μm. E, detection of the progression of peritoneal dissemination in real-time using an *in vivo* photon-counting analysis of mice treated with saline, Doc or Doc/MNTX (0.3 mg/kg). Beginning on day 7 after inoculation, the mice were divided into 4 groups based on photon counts. The mice were then treated intraperitoneally with the above reagents twice weekly until the endpoint criteria were met (middle phase).

**Fig 5:**
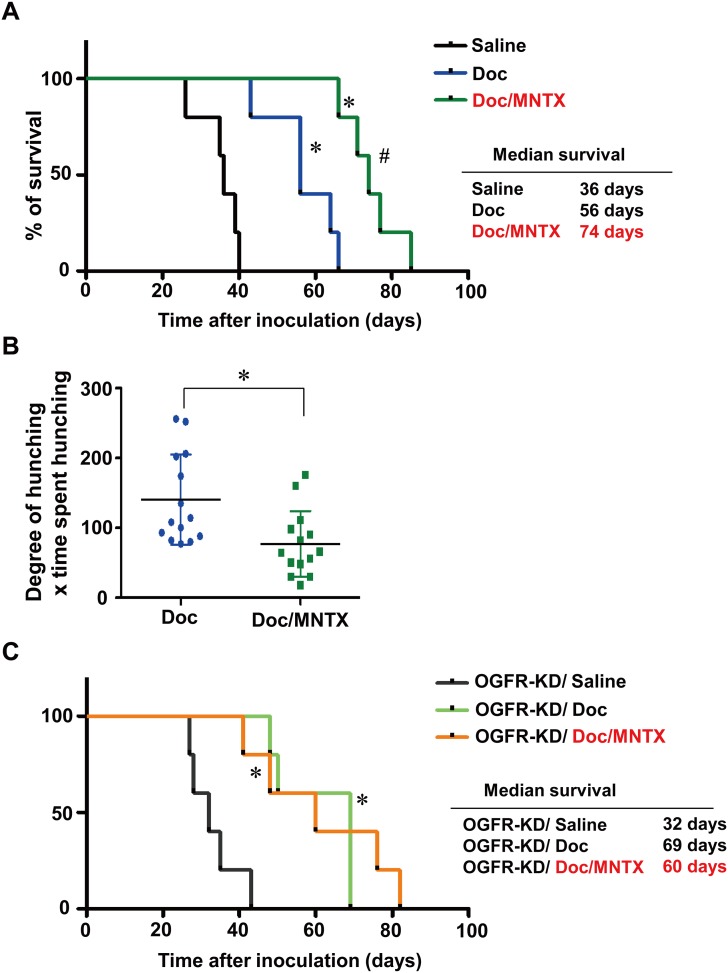
Combined use of Doc and MNTX significantly prolonged survival and alleviated abdominal pain in model mice. A, survival curves of middle-phase peritoneal metastasis model mice treated with saline, Doc, or Doc/MNTX (0.3 mg/kg) (n = 5, *p<0.05, vs. saline, #*p*<0.05, Doc vs. Doc/MNTX). Drug administration was started 7 days after the inoculation of 60As6-Luc cells. Mice were treated with Doc or a combination of Doc and MNTX 2 times a week until the endpoint criteria were met. B, visceral pain-related behavior of peritoneal metastasis model mice. Visceral pain-related behavior was assessed in terms of the degree of hunching and the time spent hunching before each drug treatment at 35 days after the inoculation of 60As6-Luc cells (mean ± SD, n = 14 each, *p<0.05). C, survival curves of peritoneal metastasis mice of *OGFR*-shRNA transfected 60As6 cells (OGFR-KD) treated with saline, Doc, or Doc/MNTX (0.3 mg/kg) (n = 5, *p<0.05, vs. saline, no significance in Doc vs. Doc/MNTX).

**Fig 6:**
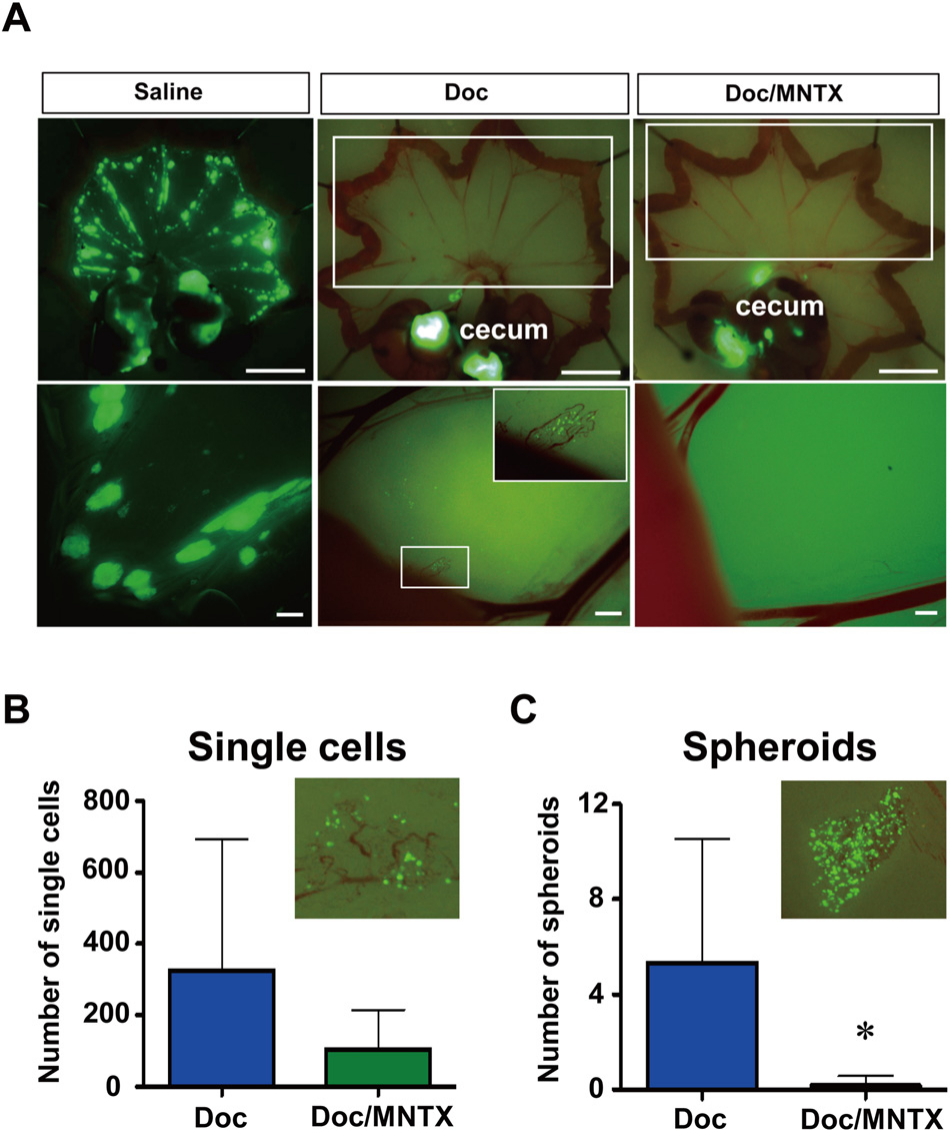
Combined use of Doc and MNTX significantly suppressed peritoneal metastasis. A, representative image of 60As6-GFP cells attached to the mesentery of saline-, Doc- and Doc/MNTX-treated mice. Drug administration started from 7 days to 35 days (saline) and 49 days (Doc or Doc/MNTX) after the inoculation of 60As6-GFP cells. Mice were treated with saline, Doc, or Doc/MNTX 2 times a week. Scale bar, upper: 10 μm, lower: 1 μm. Numbers of single cells (B) and spheroids (C) on the mesentery of Doc- and Doc/MNTX-treated mice 49 days after the inoculation of 60As6-GFP cells. Drug administration started from 7 days to 49 days after inoculation. Mice were treated with saline twice a week. (mean ± SD, n = 6 each, *p<0.05).

Conventional therapeutic modalities have failed to improve survival or outcomes among patients with peritoneal dissemination. Recently, intraperitoneal chemotherapy, which is the direct application of chemotherapeutic agents to macro/microscopic tumor seeding, has been considered to be a promising method for reducing the incidence of peritoneal dissemination [20, 21]. Therefore, we established mouse models for the intraperitoneal administration of Doc, which corresponded to 3 different phases (early, middle, and late) in the progression of peritoneal dissemination (Fig 4A–4C). The xenografts of 60As6 as well as clinical samples (Fig 1A) showed a high proportion of Ki-67-negative non-proliferating tumor cells (Fig 4D). A quite low dose of Doc (0.5 mg/kg) was used in these models, and therefore the toxicity of Doc was considered to be extremely low. Among the 3 different models, we selected the middle-phase model, which has characteristics similar to those of patients with multiple small tumor nodules that cannot be detected by a computed tomography scan. By *in vivo* imaging with luciferase-expressing 60As6 cells (60As6Luc) [22], representative chronological tumor growth was observed in the mouse peritoneal cavity treated with saline, Doc or Doc/MNTX (0.3 mg/kg) (Fig 4E). Beginning on day 7 after inoculation, the mice were divided into 4 groups based on photon counts. The mice were then treated intraperitoneally with the above reagents twice weekly until the endpoint criteria were met. In the saline-treated mice, tumor progression in the peritoneal cavity was observed on day 14 after inoculation. A marked increase in the volume of ascites was noted and moribund mice were observed around day 28. A high concentration of OGF was observed in mouse ascites (S7A Fig) as well as in the GC patients (S3B Fig). On the other hand, mice that had been treated with Doc or Doc/MNTX tended to be stable with slower tumor growth up to day 28. No ascites formation was noted in any of the mice. Consistent with these results, the intraperitoneal administration of Doc or Doc/MNTX clearly prolonged survival compared with that in saline-treated mice (Fig 5A). Notably, the survival time of Doc/MNTX-treated mice was significantly greater than that of Doc-treated mice (Fig 5A). Conversely, the combined use of Doc and OGF was likely to shorten survival relative to that with Doc alone (S7B Fig). There was no difference in survival time between MNTX-treated mice and saline-treated mice (S7C Fig). In accordance with these effects, visceral pain-related hunching behavior on day 35 after inoculation was apparently decreased in Doc/MNTX-treated mice compared with Doc-treated mice (Fig 5B). Importantly, a booster effect of MNTX with respect to Doc was not observed in an OGFR shRNA-transfectant, 60As6-OGFR-KD, *in vivo* (Fig 5C). The eradication of free tumor cells and suppression of the formation of multiple small tumor nodules on the membranes of the abdomen are major challenges in the treatment of peritoneal metastasis. To clarify the effects of Doc/MNTX in this regard, we monitored 60As6-GFP cells on the membranes of the abdomen. In mice treated with saline, several 1–3 mm tumors studding the mesentery adjacent to the bowel were observed on day 31 after inoculation (Fig 6A). In mice that had been treated with Doc or Doc/MNTX, fewer tumor nodules were noted on day 49 (Fig 6A, white box in the upper region of the cecum). However, isolated tumor cells and small tumor cell spheroids, which were located in the vascular bed of the mesentery, were found only in Doc-treated mice (Fig 6A). Based on the results of microscopic observation, there were significantly fewer single cells and spheroids in Doc/MNTX-treated mice than in Doc-treated mice (S6B and S6C Fig).

## Discussion

Peritoneal dissemination is the most frequent form of metastasis of diffuse-type GC and a leading cause of death [4, 23, 24]. Up to half of all advanced GC patients will develop peritoneal dissemination, which is already present in 5–20% of patients who are examined for potentially curative resection [25]. The five-year survival rate in patients with peritoneal dissemination is less than 3%, with overall mean and median survival periods of 6.5 and 3.1 months, respectively [26]. Although systemic chemotherapy can improve median survival up to 12 months in all advanced GC, a similar survival benefit has not been seen in patients with peritoneal dissemination due to their lower sensitivity to chemotherapy [26]. Recent reports have suggested that the molecular mechanism that is responsible for chemoresistance is based on two modalities: a tumor cell-autonomous factor and a tumor microenvironment-related factor [27]. Generally, disseminated tumor cells can regulate stromal cells, such as cancer-associated fibroblasts and mesothelial cells, in promoting the formation of ascitic fluid [28]. The ascites contains cytokines, bioactive lipids and growth factors, which can influence the proliferation of tumor cells [28]. In the present study, we found that OGF is predominantly expressed in diffuse-type GCs compared with intestinal-type GCs. The expression of OGF and its receptor OGFR is also detected in mesothelial cells and fibroblast cells. OGF is highly concentrated in the cancer-associated ascites of not only peritoneal-dissemination model mice but also GC patients. Previous studies and our current data suggest that OGF causes cell growth suppression due to p21-mediated G1-phase arrest of the cell cycle [13]. The present study demonstrated that blockade of OGF signaling by MNTX significantly increases the cell growth of diffuse-type GC cells under a low nutrient condition and/or in the co-culture system with stromal cells that spontaneously produce OGF. As shown in Fig 2C and 2D, S5E Fig, and Fig 5C, down-regulation of OGFR negated these effects of OGF and MNTX *in vitro* and *in vivo*. These results suggest that OGF/OGFR signaling is dominant in the microenvironment of diffuse-type GC, which may contribute to the dormant state of tumor cells and the lower sensitivity to anti-tumor drugs.

The present study showed that intraperitoneal treatment with Doc significantly prevents ascites formation in peritoneal dissemination model mice. In addition, isolated tumor cells and spheroids, which may be resistant to Doc, on the peritoneal surface in Doc-treated mice were clearly diminished by the co-administration of MNTX with Doc. Finally, the combined use of MNTX and Doc significantly alleviated abdominal pain and prolonged survival compared with Doc alone. Therefore, intraperitoneal chemotherapy has great potential for stopping the progression of peritoneal metastasis in patients with free tumor cells (peritoneal-lavage cytology-positive patients) or with multiple but very small tumor nodules that cannot be detected by a computed tomography scan.

In conclusion, our findings indicate that MNTX boosts the anti-tumor effect of Doc through the recycling of OGF-induced cell growth arrest, and this booster effect leads to an improved QOL and prolonged survival in peritoneal dissemination model mice. Our data propose that a strategy of awakening and killing tumor cells may have great potential for resolving the problem of chemoresistance caused by tumor cell dormancy.

## Supporting Information

S1 Table Primers for RT-PCR of human and mouse genes. (DOCX)

S1 Fig Representative histological images of diffusive-growth solid tumors, pancreatic cancer and scirrhous breast cancer. Hematoxylin-eosin, HE. Scale bar, 50 μm.
(TIF)

S2 Fig Nutrient starvation causes G1-arrest and drug resistance in diffuse-type GC cells. A, flow cytometry of 60As6 cells 72 h after transfer to serum-free medium or normal nutrient medium (containing 10% FBS). B, phase-contrast micrograph of 60As6 cells 72 h after transfer to serum-free medium or normal nutrient medium in the presence of Doc (10^-9^ and 10^-8^ M, 96 h). Scale bar, 100 μm. C, number of cells from (B). Data represent the percentages of live cells (mean ± SD, n = 3 each, *p<0.05 vs. control).
(TIF)

S3 Fig RT-PCR of *CDC6* in primary GCs. A, *CDC6* mRNA was highly expressed in most of intestinal-type GCs compared with diffuse-type GCs (upper). Some cases with a low expression of *CDC6* in intestinal-type GCs (* in upper) had rarely-proliferative cancer cells (lower). B, the amount of OGF in the ascites and peritoneal washings of GC patients. The ascites of two diffuse-type GC patients and five peritoneal washings obtained from peritoneal cytology-negative GC patients was measured by ELISA. OGF was only detected in the ascites.
(TIF)

S4 Fig Expression of *OPRM1*, *OPRD1*, *PENK* and *OGFR* in GC cell lines. A, RT-PCR analyses of *OPRM1* and *OPRD1* in HSC-60 and 60As6 cells. B, RT-PCR analyses of *PENK* and *OGFR* in several diffuse-type GC cell lines.
(TIF)

S5 Fig OGF induces G1 arrest and p21. A, cell population analysis of 60As6 cells after treatment with OGF (10^-6^–10^-4^ M) for 48 h. Different cell-cycle phases were acquired with an ArrayScan HCS Reader and separated by cell population analysis based on EdU incorporation and DNA content (mean ± SD, n = 3 each, *p<0.05). B, a cell population analysis of G1 phase after co-treatment with OGF (10^-4^ M) and MNTX (10^-5^ M) for 48 h. A population of G1 phase was analyzed based on EdU incorporation and DNA content (mean ± SD, n = 3 each, *p<0.05). C, OGF induced p21 expression. 60As6 cells were transferred to serum-free medium for 96 h to synchronize cells, and subsequently treated with OGF (10^-4^ M) for 3, 6, 9 and 12 h. Total protein was resolved by SDS-PAGE, and blotted with p21-specific antibody. D, RT-PCR and Western blotting analyses of OGFR in 60As6 cells treated with *OGFR* siRNAs or non-targeting control siRNAs. E, growth of 60As6 cells in the presence or absence of OGF (10^-4^ M) 72 h after the transfection of *OGFR* siRNA. As a control, non-targeting control siRNA was used (mean ± SD, n = 3 each, *p<0.05).
(TIF)

S6 Fig Blockade of OGF signaling by MNTX increased the growth of diffuse-type GC cells co-cultured with fibroblasts. A, RT-PCR analyses of *Penk* and *Ogfr*, fibroblast cells (NIH3T3). B, Growth of 60As6-GFP cells co-cultured with NIH3T3 cells in the presence or absence of MNTX (10^-5^ M) for 72 h. Scale bar, 20 μm. C, the growth of 60As6-GFP cells was calculated from (B) (mean ± SD, n = 3 each, *p<0.05).
(TIF)

S7 Fig Other *in vivo* experiments. A, a high concentration of OGF was observed in mouse ascites. The amount of OGF released into the ascites and peritoneal washings (PBS) obtained from mice 28 days after the inoculation of 60As6-Luc cells. The concentration of OGF was measured by ELISA. OGF was only detected in the ascites (mean ± SD, n = 5 each). B, *in vivo* effects of OGF or MNTX alone. Survival curves of middle-phase peritoneal metastasis model mice treated with saline, Doc, or Doc/OGF. Drug administration was started 7 days after the inoculation of 60As6-Luc cells. Mice were treated with Doc or a combination of Doc and OGF (10 mg/kg) 2 times a week until the endpoint criteria were met (n = 5, *p<0.05, vs. saline). C, survival curves of middle-phase peritoneal metastasis model mice treated with saline or MNTX. Mice were treated with saline or MNTX (0.3 mg/kg) 2 times a week until the endpoint criteria were met (n = 5).
(TIF)
