# Supplementary material for: Peripheral Opioid Antagonist Enhances the Effect of Anti-Tumor Drug by Blocking a Cell Growth-Suppressive Pathway In Vivo
Source: PLoS One. 2015 Apr 8;10(4):e0123407. doi: 10.1371/journal.pone.0123407 (PMC4390307; doi:10.1371/journal.pone.0123407)
Supplement: S1 Table — (DOCX) [file pone.0123407.s001.docx]

Table S1. Primers for RT-PCR of human and mouse genes

| Gene (human) | Forward | Reverse |
| --- | --- | --- |
| PENK | 5'- ACCAGCACCCTCAGAGAAA -3' | 5'- CTTGGCGAGGATCTCACTTC -3' |
| OGFR | 5'- GCCACTATAGCAGCCACCAC -3' | 5'- GGCCTTGCTTGGTATCAGAC -3' |
| ACTB | 5'- GAAGTCCCTTGCCATCCTAA -3' | 5'- GCACGAAGGCTCATCATTCA -3' |
| CDC6 | 5'- CCATGCTCAGCCATTAAGGT -3' | 5'- GGAGTGCCCAAGAAACTCAA -3' |
| OPRM1 | 5'- CGGCCAATACAGTGGATAGAA -3' | 5'- GTTAGGGCAACGGAGCAGT -3' |
| OPRD1 | 5'- CATCCACATCTTCGTCATCG -3' | 5'- AAGCAGCGCTTGAAGTTCTC -3' |
|  |  |  |
| Gene (mouse) | Forward | Reverse |
| Penk | 5'- GACAGCAGCAAACAGGATGA -3' | 5'- GTTGTCTCCCGTTCCCAGTA -3' |
| Ogfr | 5'- TCAGAGGAGTTGGCAAGGAT -3' | 5'- ACTAGGGAGAGGCCAGCACT -3' |
| Actin | 5'- CTACAAATGTGGCTGAGGAC -3' | 5'- ACAGAAGCAATGCTGTCAC -3' |
