# Supplementary figures and images for: Peripheral Opioid Antagonist Enhances the Effect of Anti-Tumor Drug by Blocking a Cell Growth-Suppressive Pathway In Vivo
Source: PLoS One. 2015 Apr 8;10(4):e0123407. doi: 10.1371/journal.pone.0123407 (PMC4390307; doi:10.1371/journal.pone.0123407)

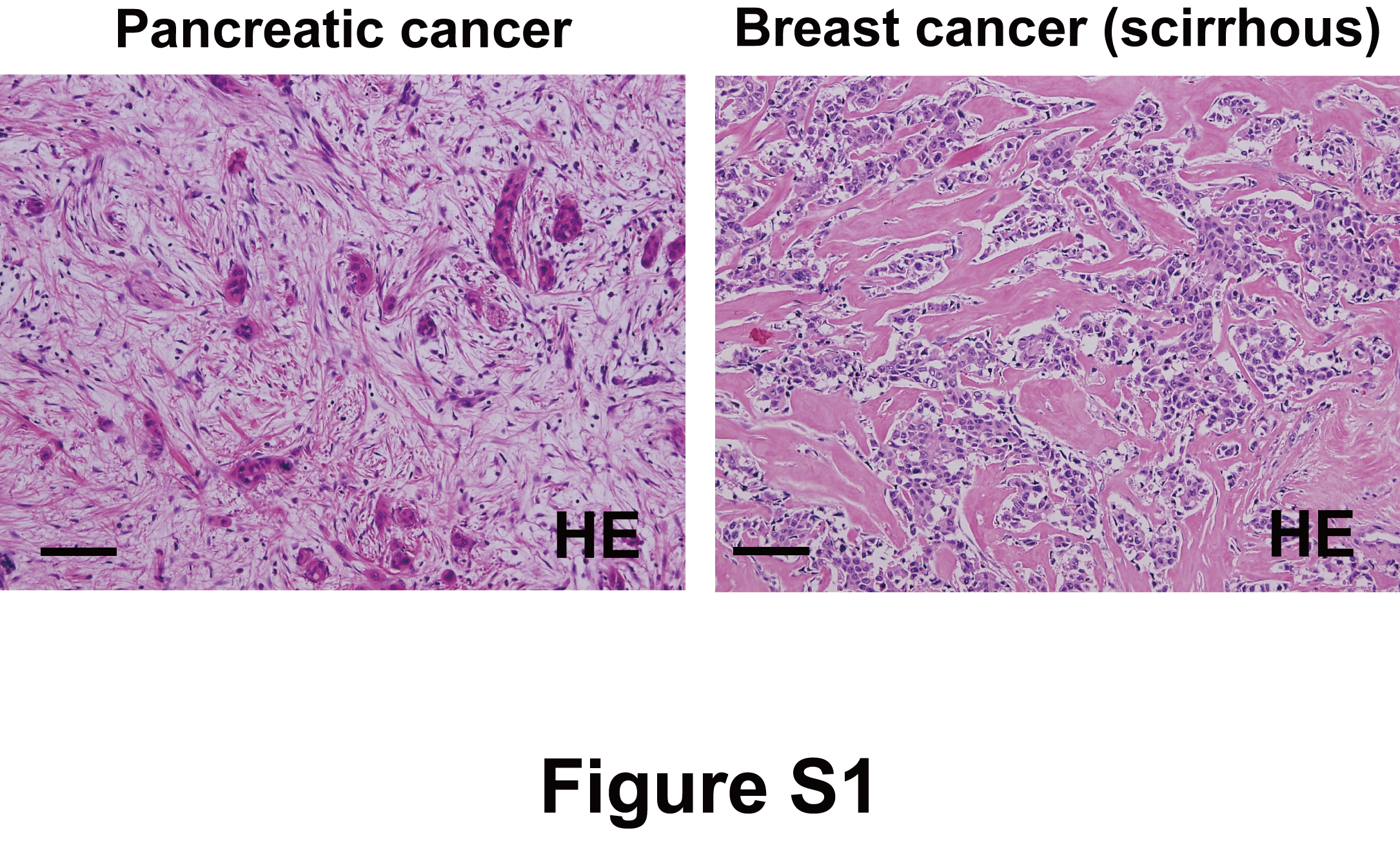

Supplement: S1 Fig — Hematoxylin-eosin, HE. Scale bar, 50 μm. (TIF) [file pone.0123407.s002.tif]

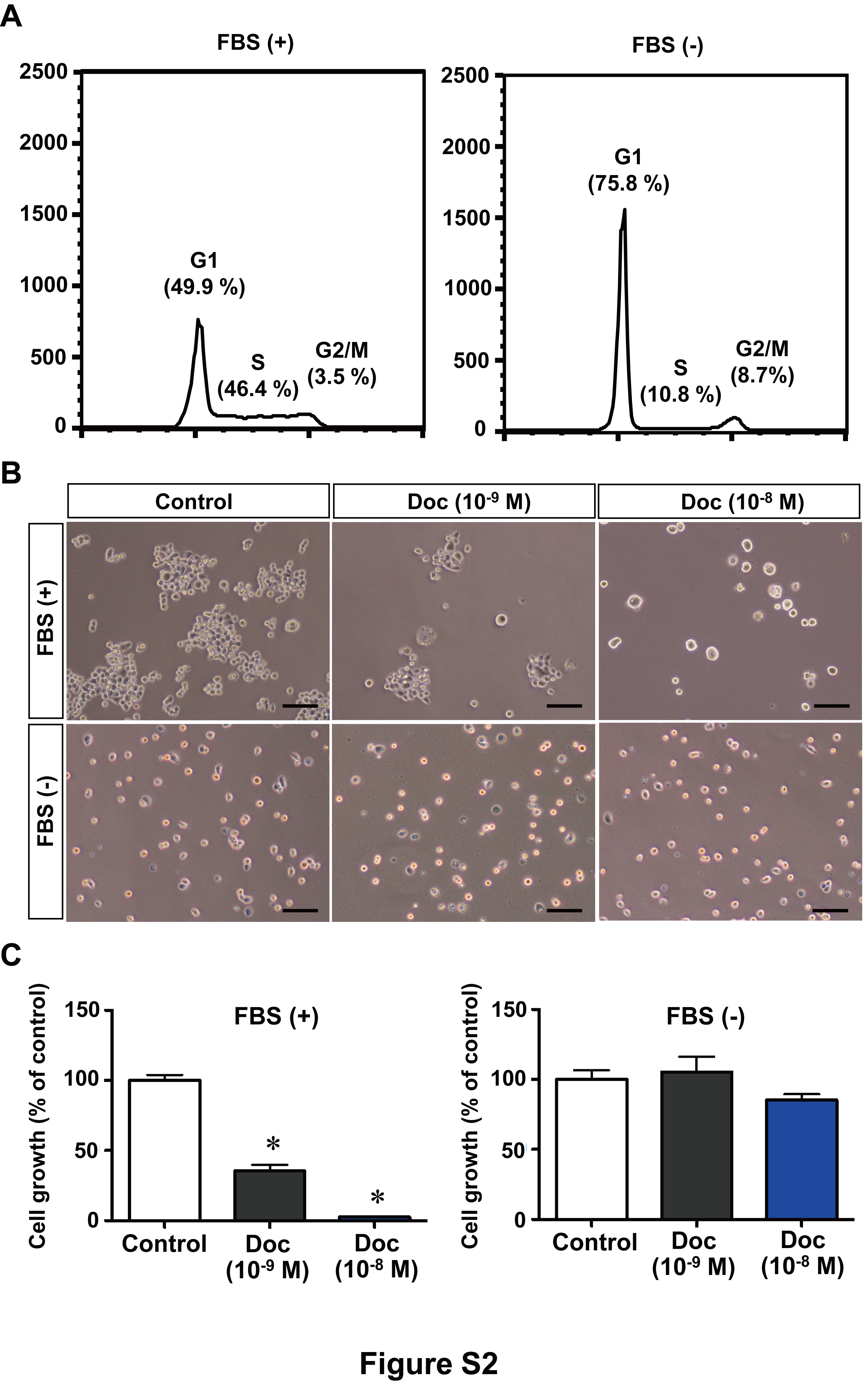

Supplement: S2 Fig — A, flow cytometry of 60As6 cells 72 h after transfer to serum-free medium or normal nutrient medium (containing 10% FBS). B, phase-contrast micrograph of 60As6 cells 72 h after transfer to serum-free medium or normal nutrient medium in the presence of Doc (10-9 and 10-8 M, 96 h). Scale bar, 100 μm. C, number of cells from (B). Data represent the percentages of live cells (mean ± SD, n = 3 each, *p<0.05 vs. control). (TIF) [file pone.0123407.s003.tif]

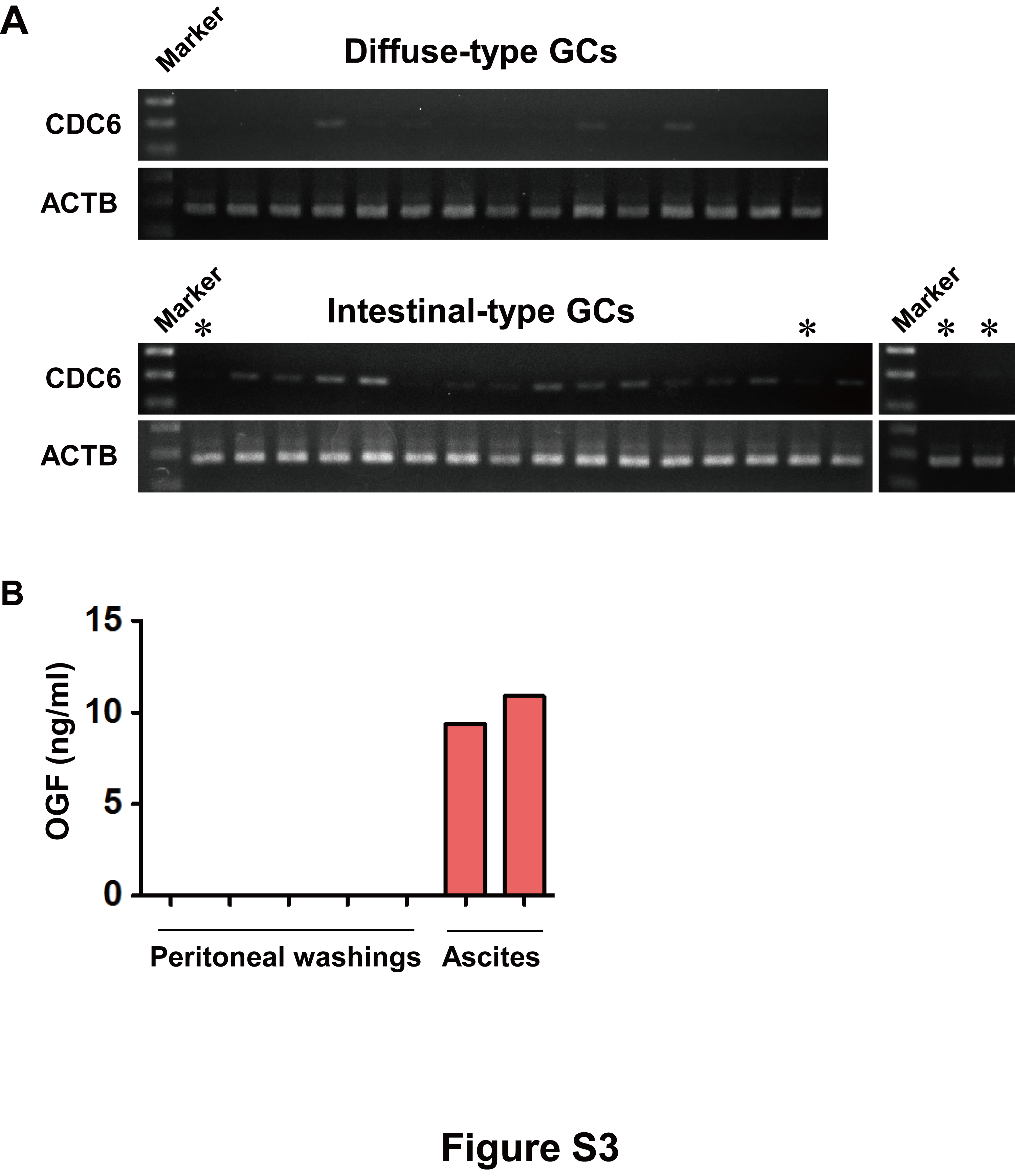

Supplement: S3 Fig — A, CDC6 mRNA was highly expressed in most of intestinal-type GCs compared with diffuse-type GCs (upper). Some cases with a low expression of CDC6 in intestinal-type GCs (* in upper) had rarely-proliferative cancer cells (lower). B, the amount of OGF in the ascites and peritoneal washings of GC patients. The ascites of two diffuse-type GC patients and five peritoneal washings obtained from peritoneal cytology-negative GC patients was measured by ELISA. OGF was only detected in the ascites. (TIF) [file pone.0123407.s004.tif]

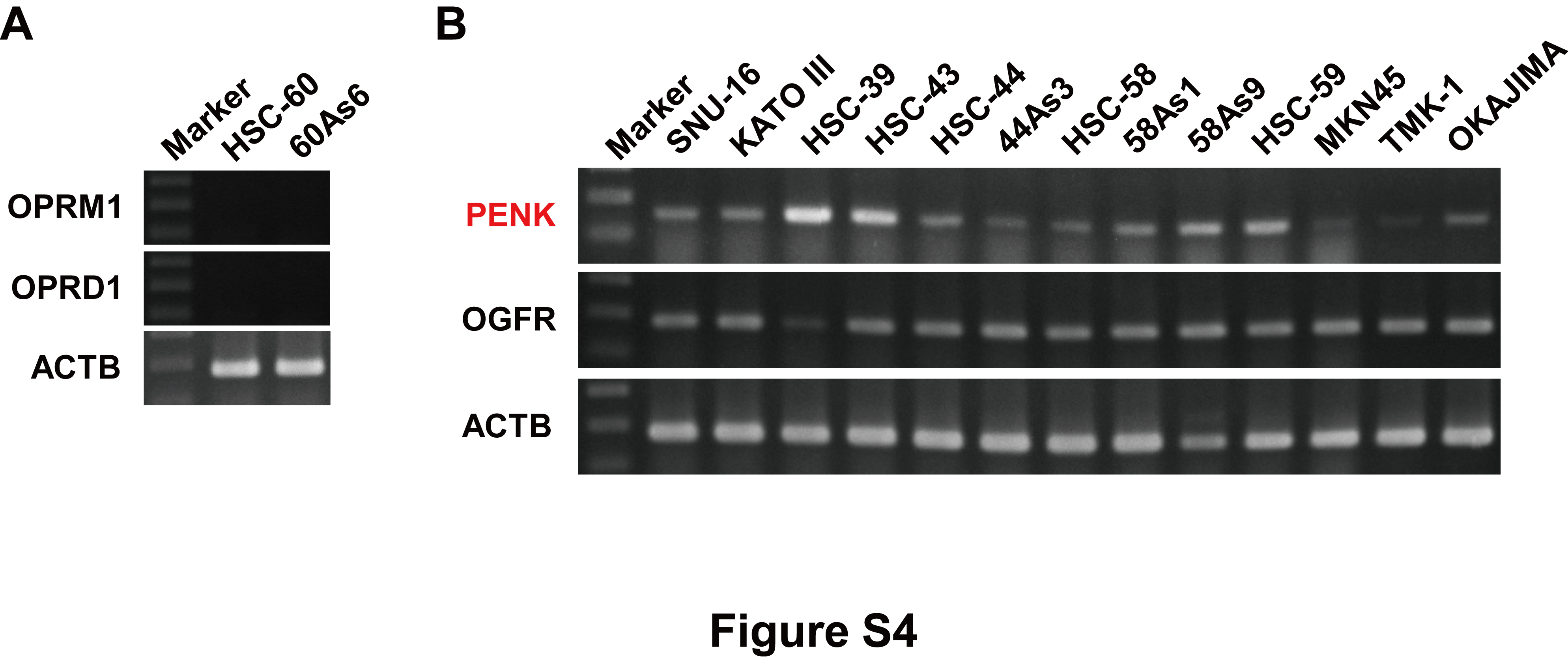

Supplement: S4 Fig — A, RT-PCR analyses of OPRM1 and OPRD1 in HSC-60 and 60As6 cells. B, RT-PCR analyses of PENK and OGFR in several diffuse-type GC cell lines. (TIF) [file pone.0123407.s005.tif]

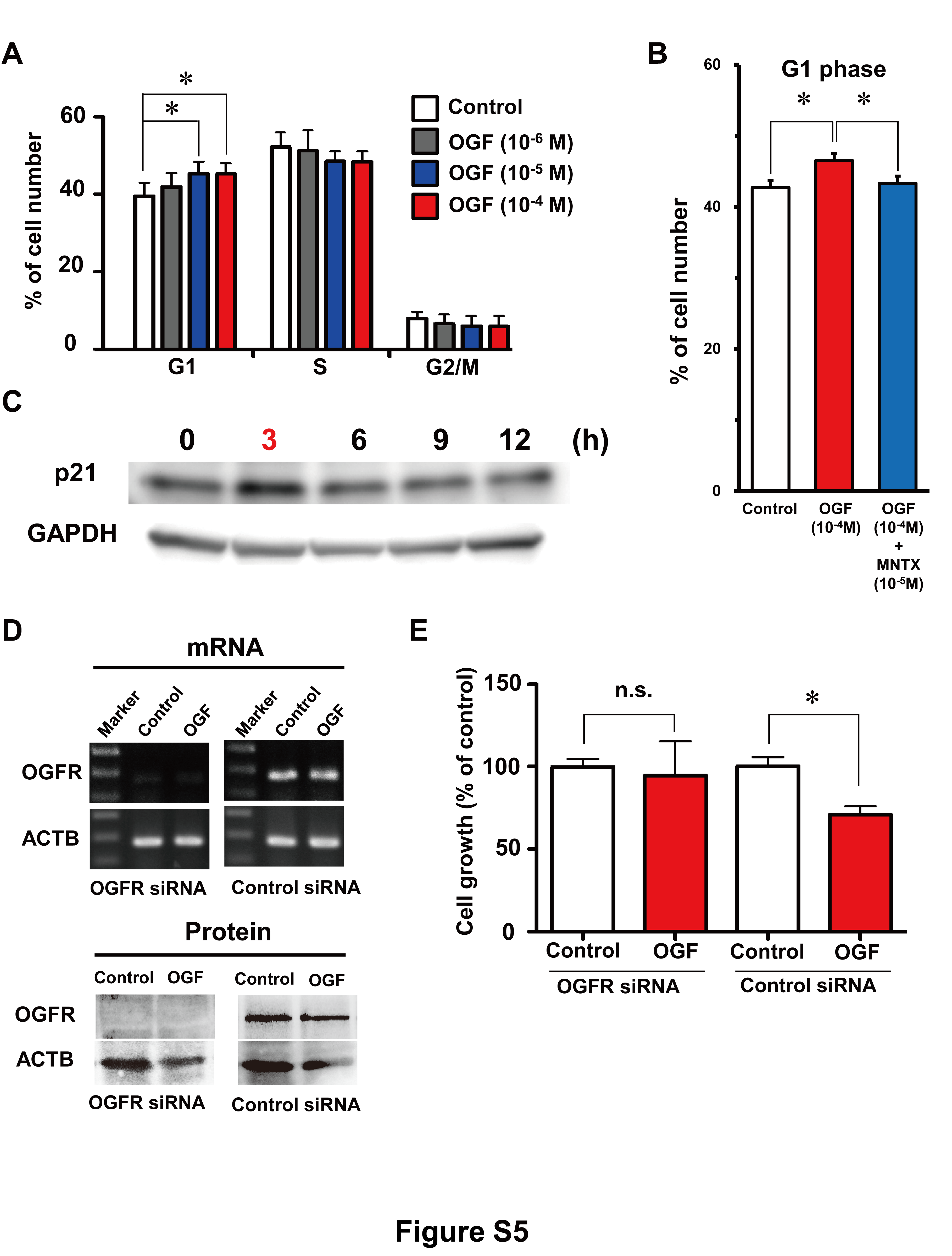

Supplement: S5 Fig — A, cell population analysis of 60As6 cells after treatment with OGF (10-6–10-4 M) for 48 h. Different cell-cycle phases were acquired with an ArrayScan HCS Reader and separated by cell population analysis based on EdU incorporation and DNA content (mean ± SD, n = 3 each, *p<0.05). B, a cell population analysis of G1 phase after co-treatment with OGF (10-4 M) and MNTX (10-5 M) for 48 h. A population of G1 phase was analyzed based on EdU incorporation and DNA content (mean ± SD, n = 3 each, *p<0.05). C, OGF induced p21 expression. 60As6 cells were transferred to serum-free medium for 96 h to synchronize cells, and subsequently treated with OGF (10-4 M) for 3, 6, 9 and 12 h. Total protein was resolved by SDS-PAGE, and blotted with p21-specific antibody. D, RT-PCR and Western blotting analyses of OGFR in 60As6 cells treated with OGFR siRNAs or non-targeting control siRNAs. E, growth of 60As6 cells in the presence or absence of OGF (10-4 M) 72 h after the transfection of OGFR siRNA. As a control, non-targeting control siRNA was used (mean ± SD, n = 3 each, *p<0.05). (TIF) [file pone.0123407.s006.tif]

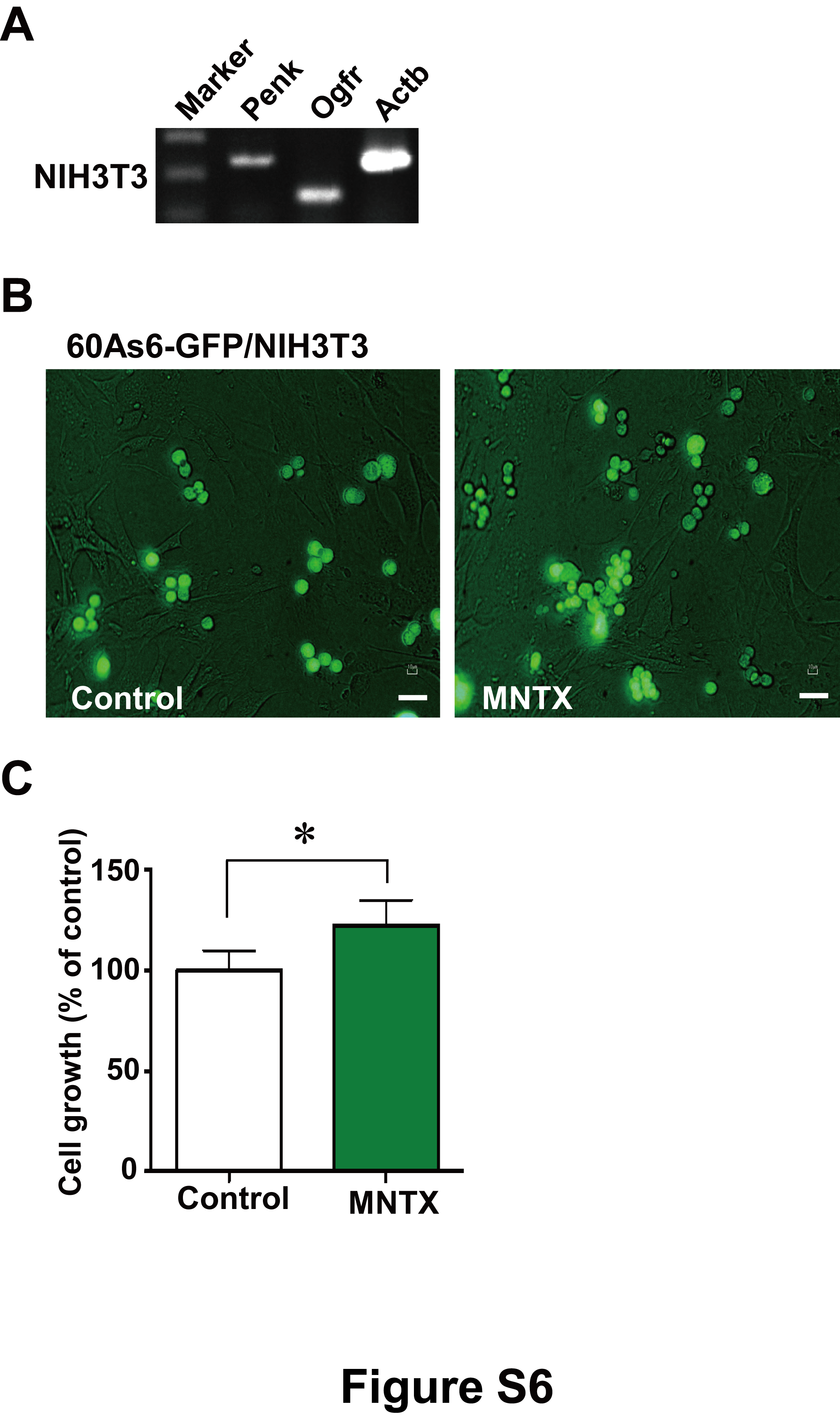

Supplement: S6 Fig — A, RT-PCR analyses of Penk and Ogfr, fibroblast cells (NIH3T3). B, Growth of 60As6-GFP cells co-cultured with NIH3T3 cells in the presence or absence of MNTX (10-5 M) for 72 h. Scale bar, 20 μm. C, the growth of 60As6-GFP cells was calculated from (B) (mean ± SD, n = 3 each, *p<0.05). (TIF) [file pone.0123407.s007.tif]

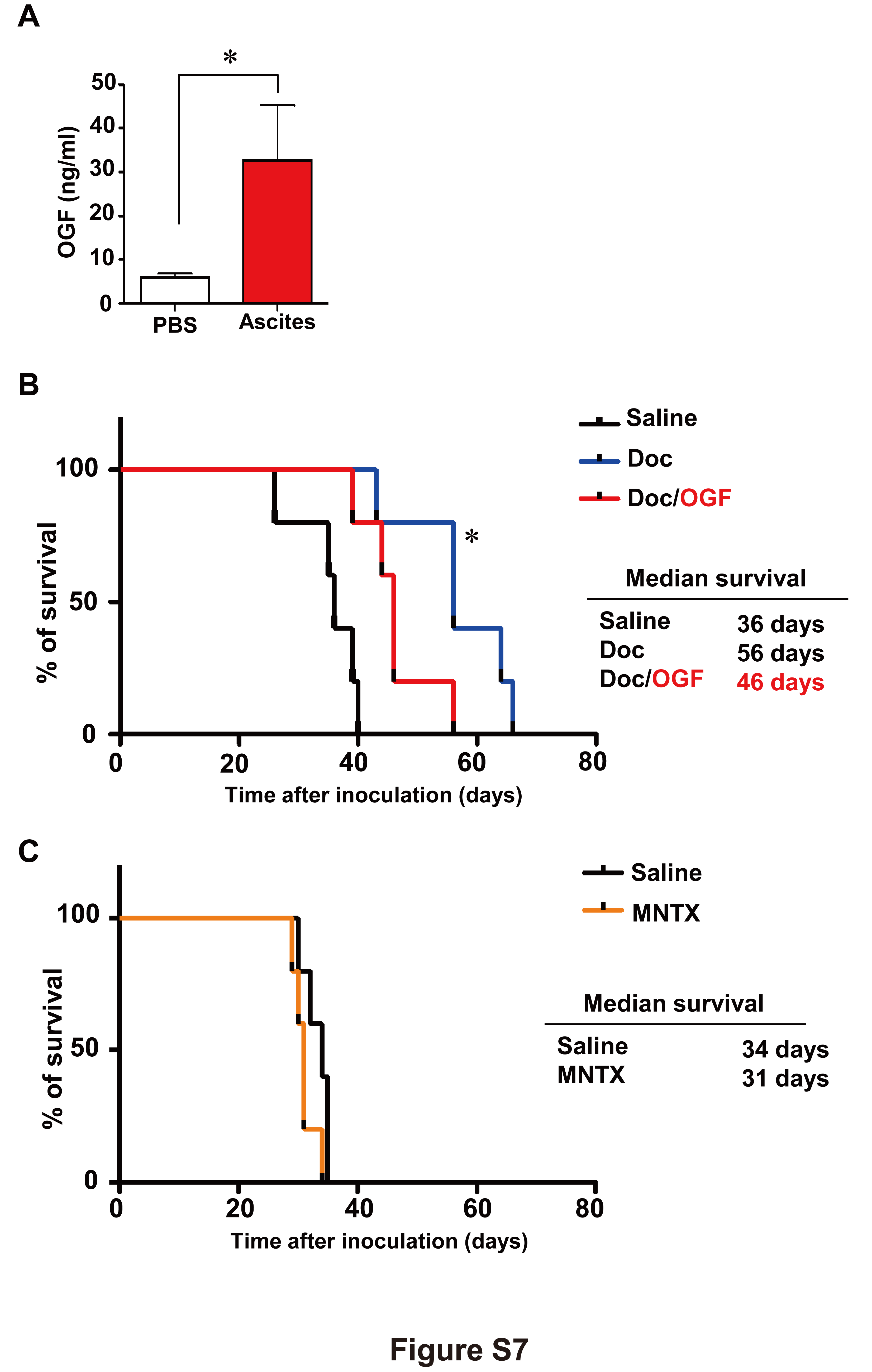

Supplement: S7 Fig — A, a high concentration of OGF was observed in mouse ascites. The amount of OGF released into the ascites and peritoneal washings (PBS) obtained from mice 28 days after the inoculation of 60As6-Luc cells. The concentration of OGF was measured by ELISA. OGF was only detected in the ascites (mean ± SD, n = 5 each). B, in vivo effects of OGF or MNTX alone. Survival curves of middle-phase peritoneal metastasis model mice treated with saline, Doc, or Doc/OGF. Drug administration was started 7 days after the inoculation of 60As6-Luc cells. Mice were treated with Doc or a combination of Doc and OGF (10 mg/kg) 2 times a week until the endpoint criteria were met (n = 5, *p<0.05, vs. saline). C, survival curves of middle-phase peritoneal metastasis model mice treated with saline or MNTX. Mice were treated with saline or MNTX (0.3 mg/kg) 2 times a week until the endpoint criteria were met (n = 5). (TIF) [file pone.0123407.s008.tif]
